# Metastatic breast cancer: Do current treatments improve quality of life? A prospective study

**DOI:** 10.1590/S1516-31802006000400006

**Published:** 2006-05-04

**Authors:** Fernanda Amado, Maria Teresa Cruz Lourenço, Daniel Deheinzelin

**Keywords:** Breast neoplasms, Neoplasm metastasis, Quality of life, Depression, Karnofsky performance status, Neoplasias mamárias, Metástase neoplásica, Qualidade de vida, Depressão, Avaliação de estado de Karnofsky

## Abstract

**CONTEXT AND OBJECTIVE:**

In metastatic breast cancer cases, the currently available therapeutic approaches provide minimal improvement in survival. As such, quality of life (QOL) becomes one of the main objectives of treatment. It is not known whether current treatments derived from trials improve QOL. The aim was to evaluate changes in QOL among metastatic breast cancer patients receiving treatment derived from trials.

**DESIGN AND SETTING:**

Prospective observational QOL survey in a tertiary cancer center.

**METHODS:**

To evaluate the influence of current treatments on patients’ QOL, the Medical Outcomes Study Short Form-36 (SF-36) and the Beck Depression Inventory (BDI) were applied on three occasions: before starting treatment and at the 6^th^ and 12^th^ weeks, to consecutive metastatic breast cancer patients over a one-year period.

**RESULTS:**

We found an improvement in QOL in the sample evaluated (n = 40), expressed by changes in the overall SF-36 score (p = 0.002) and the BDI (p = 0.004). Taken individually, the SF-36 components Pain, Social Functioning and Mental Health also improved significantly. Patients with worse initial performance status and secondary symptoms displayed greater improvement than those with better initial performance status and asymptomatic disease (p < 0.001). Patients who received more than one type of therapy showed larger gains than those given only one type (p = 0.038).

**CONCLUSIONS:**

In our environment, current metastatic breast cancer treatments can improve QOL, especially among symptomatic patients and those with low performance status.

## RESUMO

**CONTEXTO E OBJETIVO:**

As abordagens terapêuticas atuais para o câncer de mama metastático não asseguram aumento na sobrevida. Sendo assim, qualidade de vida passa a ser um dos principais objetivos do tratamento. Apesar disso, desconhecemos a influência dos tratamentos atualmente utilizados na quali-dade de vida dessa população. O objetivo do estudo foi avaliar a influência do tratamento oncológico na qualidade de vida de portadoras de câncer de mama metastático.

**TIPO DE ESTUDO E LOCAL:**

Estudo prospectivo da variação da qualidade de vida de portadoras de câncer de mama metastático tratadas em um centro médico terciário.

**MÉTODOS:**

Realizamos um levantamento prospectivo da influência dos tratamentos oncológicos, incluindo quimioterapia, tratamento hormonal, radioterapia, cirurgia e suporte clínico, na qualidade de vida de pacientes tratadas no Centro de Tratamento e Pesquisa Hospital do Câncer, em São Paulo. Utilizamos para isso o Medical Outcomes Study's Short Form-36 (SF-36) e o Inventário de Depressão de Beck (IDB), aplicados em três ocasiões: antes do início, na 6^a^ e na 12^a^ semanas após o início do tratamento.

**RESULTADOS:**

Encontramos melhora de qualidade de vida na amostra avaliada (n = 40), expressa pela variação dos índices do SF-36 (p = 0,002) e do IDB (p = 0,004). Os componentes do SF-36 que apresentaram ganhos mais significativos foram dor, aspecto social e saúde mental. Como esperado, as pacientes com piores *Performance Status* (PS) e com sintomas secundários presentes apresentaram maiores ganhos em sua qualidade de vida que as de bom PS e as assintomáticas (p < 0,001). Da mesma forma, aquelas que receberam mais de uma modalidade terapêutica em comparação com as que só receberam um tipo de tratamento (p = 0,038).

**CONCLUSÕES:**

Em nosso meio, a abordagem atual para o câncer de mama metastático mostrou-se capaz de melhorar a qualidade de vida das pacientes, especialmente das que se apresentam com sintomas secundários à doença de base ou com baixo PS.

## INTRODUCTION

Breast cancer is the form of cancer with highest incidence among women, worldwide. Women are born with a one-in-eight chance of having breast cancer during their lives. After disease recurrence, the mean survival ranges from two to three years, although it may reach up to ten years.^1,2^ During this time span, patients experience debilitating symptoms and collateral effects from the treatment.^3^ As such, metastatic breast cancer should be viewed as a chronic disease, with treatment that is essentially intended to palliate the symptoms, improve the quality of life and increase the survival.^4^

Historically, it has been difficult to demonstrate overall survival improvement in metastatic breast cancer patients. Although treatments developed recently have proven to be capable of prolonging the survival in metastatic breast cancer cases, the time span is not considerable.^5,6^ In a meta-analysis, 189 randomized clinical trials published from 1975 to 1997 were evaluated with the aim of defining the effectiveness of the medical treatment. The most important finding was the conclusion that the relevance of the results was limited by the modest survival benefit achieved, and also by the lack of assessment of how such treatments affected the patients’ quality of life, because only 9.5% of the patients were so assessed.^5^ Therefore, quality of life has become a parameter that is as important as survival, response rates and toxicity for evaluating therapeutic approaches and defining the medical procedure to be adopted.^7,8^ In this light, quality of life has been assessed in clinical trials relating to prevention^9^ and adjuvancy,^10^ and in phase III studies.^11,12^

Numerous quality-of-life evaluation instruments have been developed over recent decades, with the aim of transforming this subjective concept into an objective and measurable parameter.^13^ Some of these instruments are generic, designed for use across a wide range of chronic diseases, such as the SF-36 (Short Form-36) Health Survey, while others have been developed specifically for onco-logical patients.^14^ The SF-36 Health Survey appears to be the most suitable, in relation to other generic health status measurement tools, since it presents the best ability to discriminate between groups^15^ and has been used worldwide among cancer patients,^16–18^ thereby facilitating comparisons of health-related quality of life between independent studies.^19^ Moreover, since it is multidimensional**,** sensitive to time progression and medical interventions, and easy to apply, it is a reliable method for evaluating changes in quality of life.^20^

Notwithstanding the importance of assessing the quality of life among metastatic breast cancer patients, less than 4% of such publications mention quality of life.^21^ Because these studies, using quality of life as an outcome, usually compare two different treatment approaches, it is not known whether current metastatic breast cancer treatments derived from trials, improve quality of life. Due to lack of information on how such treatments affect the patient's quality of life, this issue remains an open question.

## OBJECTIVE

To evaluate the impact of current types of treatment on the quality of life of patients newly diagnosed with metastatic breast cancer.

## METHODS

This was a prospective observational study on the influence of current treatments on the quality of life of patients with metastatic breast cancer. The study was conducted at the Research and Treatment Center of Hospital do Câncer, a tertiary cancer center in the city of São Paulo, Brazil, from July 2001 to June 2002. This hospital provides all the types of therapy normally utilized in such cases: chemotherapy, hormonal treatment, radiotherapy, surgery and clinical support.

The study was granted approval by the local research ethics committee. Over a one-year period, consecutive breast cancer patients were considered eligible for inclusion in this study if they had a recent diagnosis of meta-static disease, had not yet started any related oncological treatment for this condition, and were able to complete the questionnaires. The Medical Outcomes SF-36 and the Beck Depression Inventory (BDI) were applied on three occasions: at base line (prior to starting treatment) and at the 6^th^ and 12^th^ weeks of treatment (± one week of leeway for the patient's convenience in scheduling appointments).

SF-36 focuses on eight different domains of health-related quality of life: physical functioning, role limitations due to physical problems, social functioning, bodily pain, general mental health, role limitations due to emotional problems, vitality and general health perceptions,^20^ and it has already been validated for populations in Brazil.^22^ The SF-36 results were calculated in accordance with the original publication: each domain scored from 0 to 100, from the worst to the best condition. The Beck Depression Inventory (BDI), comprising 21 items, scored from 0 to 3 from the best to the worst condition, was used for detection of symptoms related to depression.^23,24^

Demographic variables were collected during the patient's first interview. The clinical data collected included: time since diagnosis, disease staging and menopausal status at primary diagnosis, metastasis sites, presence of secondary symptoms and hormone receptor status. The treatment and response assessment were determined by the attending physicians, and written up in the medical records. The use of analgesics and other symptom relief medications was at the discretion of the physician in charge. The patient's Eastern Cooperative Oncology Group (ECOG) performance status was rated at each interview.^25^

Treatments were classified as follows: surgery, considered to be any invasive approach related to the metastatic sites, but not including procedures only used for diagnosis; chemotherapy, including the use of anti-cancer drugs administered by any means; hormonal treatment, i.e. chemical endocrine therapy; radiotherapy, including any form of application; and finally, clinical support, defined as any other medical intervention not included in the preceding items. The treatment was classified as support only when no other therapeutic approach was employed.

For statistical analysis, the Friedman Test was used to compare the SF-36 and the BDI scores. Analysis of variance (ANOVA) was utilized to assess the effects of clinical characteristics, treatments and treatment response on the SF-36 and BDI scores (two-way ANOVA). The significance level was set at 0.05.

## RESULTS

Over this one-year period, 48 consecutive cases with diagnoses of metastatic breast cancer were enrolled in the study. One patient who died prior to the second interview, two who underwent treatment in other institutions and five that did not attend the third interview were excluded from the study. Thus, 40 patients accomplished the program of three interviews that had been envisaged. The second interview was held between 25 and 46 days after treatment started, with a mean of 36 days (5.14 weeks), and the third was held between 66 and 88 days after treatment started, with a mean of 79 days (11.29 weeks).

Significant positive changes in quality of life and depression-related symptoms were observed (Table 1). Three subitems of the SF-36, pain (48.7 versus 68.4, p = 0.001), social functioning (57.8 versus 71.2, p = 0.042) and mental health (60.5 versus 75.5, p < 0.001), showed significant improvement. Overall, the SF-36 mental score also presented significant change (228.2 versus 283.8, p = 0.005).

**Table 1.** Changes in scores over time* on the Short Form-36 (SF-36) and Beck Depression Inventory (BDI) questionnaires among women diagnosed with metastatic breast cancer (n = 40)

Variable	First evaluation mean ± SD	Second evaluation mean ± SD	Third evaluation mean ± SD	P†
SF-36	438.6 ± 178.1	479.5 ± 168.1	527.6 ± 182.0	0.002
Total physical score	210.4 ± 103.3	223.7 ± 98.1	243.8 ± 109.3	0.058
Physical functioning	66.9 ± 33.1	65.9 ± 33.3	68.0 ± 33.0	0.216
Physical role	33.1 ± 44.0	35.0 ± 43.0	44.4 ± 46.2	0.230
Bodily pain	48.7 ± 33.5	58.8 ± 28.6	68.4 ± 28.9	0.001
General health	61.7 ± 22.0	64.0 ± 23.0	63.0 ± 26.7	0.792
Total mental score	228.2 ± 98.2	255.8 ± 90.1	283.8 ± 89.8	0.005
Vitality	53.2 ± 26.5	59.9 ± 22.3	65.4 ± 23.1	0.057
Social functioning	57.8 ± 32.2	63.1 ± 33.1	71.2 ± 32.6	0.042
Emotional role	56.7 ± 46.0	62.5 ± 46.0	71.7 ± 42.4	0.274
Mental health	60.5 ± 25.4	70.3 ± 20.8	75.5 ± 18.3	< 0.001
BDI	13.1 ± 9.8	11.5 ± 7.5	9.8 ± 8.1	0.004

*SD = standard deviation;*

* *SF-36 changes positively with improvement, whereas BDI changes inversely;*

† *Friedman test.*

The characteristics of the patients, the disease and the treatments administered and their interference with quality of life are presented in Table 2.

**Table 2.** Change in quality of life on Short Form-36 questionnaire according to demographic, disease and treatment characteristics among metastatic breast cancer patients, in three different evaluations over 12 weeks (n = 40, ANOVA)

Variable	n	First evaluation mean ± SD	Second evaluation mean ± SD	Third evaluation mean ± SD	P
Age					
< 50 years old	19	401.5 ± 152.1	425.3 ± 142.6	535.5 ± 174.2	0.294
> 50 years old	21	472.3 ± 196.3	528.4 ± 177.5	520.5 ± 192.8	
Partner					
no	20	401.9 ± 187.9	471.0 ± 188.7	495.8 ± 186.8	0.308
yes	20	475.4 ± 164.2	487.9 ± 149.2	559.5 ± 176.0	
Educational level					
grade school or less*	17	360.5 ± 168.0	434.5 ± 176.8	483.3 ± 171.1	0.053
high school or more	23	496.4 ± 165.8	512.6 ± 157.0	560.3 ± 186.6	
Labor income					
no*	21	470.8 ± 189.7	543.2 ± 144.3	567.9 ± 160.3	0.054
yes	19	403.1 ± 161.9	409.0 ± 167.6	483.0 ± 198.1	
Performance status					
0	10	645.6 ± 91.8	627.2 ± 132.6	662.9 ± 106.7	< 0.001†
1	16	448.0 ± 125.2	485.8 ± 141.0	549.2 ± 171.2	
2	10	320.8 ± 79.2	392.7 ± 152.6	452.4 ± 159.3	
3	2	114.2 ± 93.7	328.5 ± 78.5	229.0 ± 186.7	
4	2	242.7 ± 72.5	274.5 ± 77.1	353.0 ± 24.8	
Comorbidity					
no	26	469.7 ± 155.6	498.0 ± 150.3	559.6 ± 152.0	0.139
yes*	14	380.9 ± 207.6	445.0 ± 198.3	468.2 ± 221.6	
Disease-free interval					
< 2 years	14	413.8 ± 195.5	438.5 ± 178.4	496.9 ± 187.3	0.350
> 2 years	26	452.2 ± 170.5	501.5 ± 161.5	544.1 ± 180.7	
Estrogen receptor					
+/unknown	23	443.6 ± 187.6	499.6 ± 170.2	564.2 ± 173.1	0.334
–	17	432.0 ± 170.0	452.1 ± 166.4	478.1 ± 187.2	
Metastatic sites					
1	22	462.1 ± 184.4	479.0 ± 164.5	524.8 ± 180.3	0.770
> 1	18	410.0 ± 171.30	480.0 ± 177.2	531.1 ± 189.3	
Metastases					
not visceral	18	453.0 ± 203.5	478.4 ± 165.3	546.4 ± 164.8	0,703
visceral	22	426.9 ± 158.4	480.3 ± 174.2	512.2 ± 197.5	
Symptoms					
no	7	675.6 ± 84.6	676.9 ± 83.7	693.6 ± 83.0	< 0.001
yes*	33	388.4 ± 149.8	437.6 ± 151.0	492.4 ± 178.3	
Treatment types					
1	28	479.8 ± 168.6	511.7 ± 169.2	554.7 ± 188.3	0.038
> 1*	12	342.7 ± 168.6	404.3 ± 145.4	464.4 ± 155.7	

*ANOVA = analysis of variance; SD = standard deviation;*

* *greater positive change in quality of life;*

† *the worse the performance status, the greater the change in quality of life.*

Presence of symptoms secondary to metastatic disease was a determining factor with regard to significant gain in quality of life following treatment. Other characteristics of the disease were not determinants of change in quality of life. There was a significant statistical difference between patients who underwent only one treatment and those given more than one type, with overall responses greater in the latter group. The patients’ individual characteristics were unrelated to the change in the quality of life, except for the initial performance status, which presented an inverse correlation with the gain in quality of life (Table 2).

## DISCUSSION

Analysis of our data showed that onco-logical treatment might improve the quality of life for metastatic breast cancer patients. Improvement was observed particularly in characteristics relating to pain, social functioning and mental health. The influence of pain on the quality of life of oncology patients has already been established: quality of life is inversely proportional to the severity of the pain experienced by patients.^26^ In a study relating to lung cancer, there was significant deterioration in functional status and quality of life caused by chronic pain, secondary to the surgical resection procedure utilized.^27^ Although in the present study the pain levels and control measures adopted were not evaluated, our results show that the SF-36 pain scale had a strong influence on the change in the patients’ quality of life over the course of the treatment, thus showing the importance of pain control in this population. Therefore, if oncological treatment is capable of controlling pain, the observed improvement in quality of life was not unexpected.

The BDI revealed symptoms of anxiety or depression in 26.2% of the interviewees, twelve weeks after they had received their diagnoses of metastatic disease, which is similar to reported rates.^28^ The significant changes in the SF-36 mental score seen in our sample, together with the changes in the same direction and of similar magnitude shown by applying the BDI, substantiate the influence of metastatic breast cancer and the consequences of its treatment on the psychosocial characteristics of these patients.

Our study has shown that oncological treatment has the potential to enhance the quality of life of metastatic breast cancer patients, provided that they present symptomatic disease that does not have any proven prognostic value in relation to breast cancer. This finding is in accordance with previous studies.^29^ In 1996, the Outcomes Working Group of the American Society of Clinical Oncology stated that the improvement in quality of life for patients responding to treatment would probably be related to the fact that they presented symptoms secondary to metastatic disease.^8^

In spite of the advances in diagnosis and treatment seen over the last two decades, no consistent benefit regarding survival has been shown through treatment of metastatic disease in asymptomatic breast cancer patients.^30^

This suggests that follow-up programs based on regular physical examinations and yearly mammography alone are as effective as the more intensive approaches based on regularly performing laboratory tests and using other diagnostic tools.^31,32^ The lack of increase in quality of life through treatment in such cases in the present study corroborates the idea that early diagnosis of asymptomatic metastatic breast disease is not advantageous. On the other hand, since there was a major improvement in quality of life among symptomatic patients, treatment is justified even if it does not change survival.^30^

The quality of life among patients with the poorest performance status is benefited more through treatment than among those with better performance status. Several studies have related performance status to quality of life,^33–36^ but only one investigated the relationship between the initial performance status and the change in quality of life, reaching a conclusion similar to ours.^37^ This statistically significant finding suggests that there needs to be a change in the procedure adopted by physicians. Nowadays, such patients are usually excluded from clinical trials, but the population with a precarious performance status seems to be the one that would benefit more from the introduction of treatment.

We found that patients given more than one type of therapy achieved better quality of life than did those given a single type. This can be easily understood if we note that in metastatic breast cancer cases, surgery and radiotherapy are usually associated with systemic treatments (chemotherapy or hormonal treatment), with the aim of immediately resolving localized intercurrences such as fluid drainage into cavities and correction of pathological fractures, bone pains and compression phenomena that are generally accompanied by debilitating symptoms that need to be speedily palliated. Indeed, in our sample, only the patients presenting symptoms secondary to the metastatic disease were given more than one type of treatment.

Of course the major limitation of the present study was the limited number of patients enrolled and the short follow-up period. The majority of larger studies use performance status, tumor response and toxicity as surrogates for the patients’ quality of life, thereby portraying a unilateral view of the events assessed.^3,21^ Adoption of a parameter that really evaluates the patients’ perceptions in randomized studies and in clinical practice would provide a more realistic understanding of the effects of medical interventions on patients. Quality-of-life measurements have the capability to depict patients’ physical, psychological and social characteristics, as an independent, first-priority parameters. They make it possible to justify treatments for metastatic breast cancer cases, even if survival is not prolonged.^8^ As a new parameter, quality of life must be explored in order to reveal its potential as a prognostic factor and as an indicator of response to treatment, thus helping to define when and how such patients should be treated.

## CONCLUSIONS

Our study has shown that current onco-logical treatment has the potential to enhance the quality of life of metastatic breast cancer patients, provided that they present symptomatic disease, whereas there is no gain for asymptomatic patients. The poorer the patient's initial performance status, the greater will be the change in the quality of life, which therefore encourages oncological treatment for low performance status populations. Improvement in the quality of life, when found, occurs more in relation to psychosocial characteristics than to physical ones. These findings need to be substantiated by other studies.

## References

[1] McPherson K, Steel CM, Dixon JM (2000). ABC of breast diseases. Breast cancer-epidemiology, risk factors, and genetics. BMJ.

[2] Feuer EJ, Wun LM, Boring CC, Flanders WD, Timmel MJ, Tong T (1993). The lifetime risk of developing breast cancer. J Natl Cancer Inst.

[3] Carlson RW (1998). Quality of life issues in the treatment of metastatic breast cancer. Oncology (Williston Park).

[4] Cardoso F, Di LA, Lohrisch C, Bernard C, Ferreira F, Piccart MJ (2002). Second and subsequent lines of chemotherapy for metastatic breast cancer: what did we learn in the last two decades?. Ann Oncol.

[5] Fossati R, Confalonieri C, Torri V (1998). Cytotoxic and hormonal treatment for metastatic breast cancer: a systematic review of published randomized trials involving 31,510 women. J Clin Oncol.

[6] Ghersi D, Wilcken N, Simes J, Donoghue E (2005). Taxane containing regimens for metastatic breast cancer. Cochrane Database Syst Rev.

[7] Brady MJ, Cella DF, Mo F (1997). Reliability and validity of the Functional Assessment of Cancer Therapy-Breast quality-of-life instrument. J Clin Oncol.

[8] (1996). Outcomes of cancer treatment for technology assessment and cancer treatment guidelines. American Society of Clinical Oncology. J Clin Oncol.

[9] Day R, Ganz PA, Costantino JP, Cronin WM, Wickerham DL, Fisher B (1999). Health-related quality of life and tamoxifen in breast cancer prevention: a report from the National Surgical Adjuvant Breast and Bowel Project P-1 Study. J Clin Oncol.

[10] Ganz PA, Rowland JH, Meyerowitz BE, Desmond KA (1998). Impact of different adjuvant therapy strategies on quality of life in breast cancer survivors. Recent Results Cancer Res.

[11] Geels P, Eisenhauer E, Bezjak A, Zee B, Day A (2000). Palliative effect of chemotherapy: objective tumor response is associated with symptom improvement in patients with metastatic breast cancer. J Clin Oncol.

[12] Norris B, Pritchard KI, James K (2000). Phase III comparative study of vinorelbine combined with doxorubicin versus doxorubicin alone in disseminated metastatic/recurrent breast cancer: National Cancer Institute of Canada Clinical Trials Group Study MA8. J Clin Oncol.

[13] Fallowfield L (2002). Quality of life: a new perspective for cancer patients. Nat Rev Cancer.

[14] Langenhoff BS, Krabbe PF, Wobbes T, Ruers TJ (2001). Quality of life as an outcome measure in surgical oncology. Br J Surg.

[15] Essink-Bot ML, Krabbe PF, Bonsel GJ, Aaronson NK (1997). An empirical comparison of four generic health status measures. The Nottingham Health Profile, the Medical Outcomes Study 36-item Short-Form Health Survey, the COOP/WONCA charts, and the EuroQol instrument. Med Care.

[16] Cooley ME, McCorkle R, Knafl GJ (2005). Comparison of health-related quality of life questionnaires in ambulatory oncology. Qual Life Res.

[17] Okamoto T, Shimozuma K, Katsumata N (2003). Measuring quality of life in patients with breast cancer: a systematic review of reliable and valid instruments available in Japan. Breast Cancer.

[18] Mosconi P, Apolone G, Barni S, Secondino S, Sbanotto A, Filiberti A (2002). Quality of life in breast and colon cancer long-term survivors: an assessment with the EORTC QLQ-C30 and SF-36 questionnaires. Tumori.

[19] Yost KJ, Haan MN, Levine RA, Gold EB (2005). Comparing SF-36 scores across three groups of women with different health profiles. Qual Life Res.

[20] Ware JE (2000). SF-36 health survey update. Spine.

[21] Mosconi P, Colozza M, De Laurentiis M, De Placido S, Maltoni M (2001). Survival, quality of life and breast cancer. Ann Oncol.

[22] Ciconelli RM, Ferraz MB, Santos W, Meinão I, Quaresma MR (1999). Tradução para a língua portuguesa e validação do questionário genérico de avaliação de qualidade de vida SF-36 (Brasil SF-36). [Brazilian-Portuguese version of the SF-36. A reliable and valid quality of life outcome measure]. Rev Bras Reumatol.

[23] Gorenstein C, Andrade L (1996). Validation of a Portuguese version of the Beck Depression Inventory and the State-Trait Anxiety Inventory in Brazilian subjects. Braz J Med Biol Res.

[24] Beck AT, Ward CH, Mendelson M, Mock J, Erbaugh J (1961). An inventory for measuring depression. Arch Gen Psychiatry.

[25] Conill C, Verger E, Salamero M (1990). Performance status assessment in cancer patients. Cancer.

[26] Wang XS, Cleeland CS, Mendoza TR (1999). The effects of pain severity on health-related quality of life: a study of Chinese cancer patients. Cancer.

[27] Handy JR, Asaph JW, Skokan L (2002). What happens to patients undergoing lung cancer surgery? Outcomes and quality of life before and after surgery. Chest.

[28] Bottomley A (1998). Depression in cancer patients: a literature review. Eur J Cancer Care (Engl).

[29] Osoba D (1994). Lessons learned from measuring health-related quality of life in oncology. J Clin Oncol.

[30] Pivot X, Asmar L, Hortobagyi GN, Theriault R, Pastorini F, Buzdar A (2000). A retrospective study of first indicators of breast cancer recurrence. Oncology.

[31] Rojas MP, Telaro E, Russo A (2005). Follow-up strategies for women treated for early breast cancer. Cochrane Database Syst Rev.

[32] Mollick JA, Carlson RW (2004). Rational surveillance programs for early stage breast cancer patients after primary treatment. Breast Dis.

[33] Coates A, Gebski V, Signorini D (1992). Prognostic value of quality-of-life scores during chemotherapy for advanced breast cancer. Australian New Zealand Breast Cancer Trials Group. J Clin Oncol.

[34] Hann DM, Jacobsen PB, Martin SC, Kronish LE, Azzarello LM, Fields KK (1997). Quality of life following bone marrow transplantation for breast cancer: a comparative study. Bone Marrow Transplant.

[35] Murray KJ, Nelson DF, Scott C (1995). Quality-adjusted survival analysis of malignant glioma. Patients treated with twice-daily radiation (RT) and carmustine: a report of Radiation Therapy Oncology Group (RTOG) 83-02. Int J Radiat Oncol Biol Phys.

[36] Sprangers MA, Groenvold M, Arraras JI (1996). The European Organization for Research and Treatment of Cancer breast cancer-specific quality-of-life questionnaire module: first results from a three-country field study. J Clin Oncol.

[37] Greimel E, Thiel I, Peintinger F, Cegnar I, Pongratz E (2002). Prospective assessment of quality of life of female cancer patients. Gynecol Oncol.

